# Clinical verification of sensitivity to preoperative chemotherapy in cases of androgen receptor-expressing positive breast cancer

**DOI:** 10.1038/bjc.2015.434

**Published:** 2016-01-12

**Authors:** Yuka Asano, Shinichiro Kashiwagi, Naoyoshi Onoda, Kento Kurata, Tamami Morisaki, Satoru Noda, Tsutomu Takashima, Masahiko Ohsawa, Seiichi Kitagawa, Kosei Hirakawa

**Affiliations:** 1 Department of Surgical Oncology, 1-4-3 Asahi-machi, Abeno-ku, Osaka 545-8585, Japan; 2 Department of Diagnostic Pathology, 1-4-3 Asahi-machi, Abeno-ku, Osaka 545-8585, Japan; 3 Department of Physiology, Osaka City University Graduate School of Medicine, 1-4-3 Asahi-machi, Abeno-ku, Osaka 545-8585, Japan

**Keywords:** triple-negative breast cancer, androgen receptor, pathological complete response, luminal androgen receptor, intrinsic subtype

## Abstract

**Background:**

Triple-negative breast cancer (TNBC) patients testing positive for androgen receptor (AR) expression are thought to be chemotherapy resistant, similar to other hormone receptor-positive breast cancers; however, this has not been substantially validated in the clinic. In this study, we investigated the association between chemotherapy sensitivity and AR expression in patients treated with neoadjuvant chemotherapy (NAC) using standardised chemotherapy criteria and regimens.

**Methods:**

A total of 177 patients with resectable early-stage breast cancer were treated with NAC. Oestrogen receptor, progesterone receptor, HER2, Ki67 and AR status were assessed immunohistochemically.

**Results:**

Sixty-one patients were diagnosed with TNBC; AR expression was identified in 23 (37.7%), which was significantly less common than that found in non-TNBC patients (103 of 116; 88.8% *P*<0.001). The rate of pathological complete response after NAC was significantly lower (*P*=0.001), and disease recurrence was more common (*P*=0.008) in patients with AR-positive compared with those with AR-negative TNBC. In TNBC cases, as expected, the non-recurrence period in cases that were negative for AR expression was significantly extended (*P*=0.006, log-rank).

**Conclusions:**

Androgen receptor expressions may be useful as biomarkers to predict treatment responses to NAC in TNBC. Moreover, induction of a change in subtype to the AR-negative phenotype was observed after NAC.

Breast cancer is a typical hormone-dependent malignant tumour. Expression of the oestrogen receptor (ER) is frequently observed in breast cancer and plays a central role in disease development and progression. In addition, expression of the androgen receptor (AR) has also been frequently noted, suggesting that androgens may also play a role in breast cancer biodynamics (Soreide *et al*, 1992; Isola, 1993; Kuenen-Boumeester *et al*, 1996; Brys *et al*, 2002; Moinfar *et al*, 2003; Ogawa *et al*, 2008). Androgen receptor expression has been identified in 70–90% of breast tumours, similar to the frequency of ER expression (Hall and Solehdin, 1996; Kuenen-Boumeester *et al*, 1996). Although previous reports indicate that androgens inhibit the progression of breast cancer (Poulin *et al*, 1988; de Launoit *et al*, 1991; Ando *et al*, 2002), the precise mechanisms and clinical significance of AR in breast cancer remain unclear.

The diversity of breast cancer subtypes is exemplified by the differential sensitivity of various individuals to chemotherapy. Genetic analysis using cDNA microarrays revealed that breast cancer may be categorised into multiple groups based on clinical differences: five breast cancer intrinsic subtypes (luminal A, luminal B, human epidermal growth factor receptor type (HER) 2 enriched, claudin-low, basal like) and a normal breast-like group (Perou *et al*, 2000; Abd El-Rehim *et al*, 2005; Mattie *et al*, 2006; Prat and Perou, 2011). Among these, the basal-like subtype exhibits characteristics similar to myoepithelial/basal cells, with many cases consistent with triple-negative breast cancer (TNBC), in that they are immunohistochemically ER negative, progesterone receptor (PgR) negative and HER2 negative. For this reason, endocrine therapy and anti-HER2 therapy are unlikely to be effective, leaving chemotherapy as the only alternative (Sorlie *et al*, 2001, 2003; Nielsen *et al*, 2004; Rakha *et al*, 2006; Bauer *et al*, 2007).

Given the lack of treatment choices and the biological attributes of TNBC, this type of breast cancer generally has a poor prognosis, and new therapeutic targets are being investigated (Lehmann *et al*, 2011; Metzger-Filho *et al*, 2012; Masuda *et al*, 2013). Previous studies indicate that AR expression could be a potential target for anti-androgen therapy in TNBC (Safarpour and Tavassoli, 2014). Similarly, AR-expressing TNBC has been considered chemotherapy resistant from observations from preclinical experiments (Graham *et al*, 2010; Lehmann *et al*, 2011), similar to ER-positive breast cancer. However, few studies have verified the efficacy of chemotherapy in patients with AR-positive TNBC at the clinical level. In neoadjuvant chemotherapy (NAC) cases, we hypothesised that the pathological complete response (pCR) rate would decrease if AR-positive TNBC showed chemoresistance.

In the present study, we retrospectively investigated the outcomes of NAC in patients with TNBC, using standardised criteria and regimens. The aim of this study was to clarify the differences in chemosensitivity, clinically and pathologically, based on AR expression in patients with breast cancer.

## Materials and methods

### Patients

A total of 177 patients with resectable early-stage breast cancer diagnosed as stage IIA (T1, N1, M0 or T2, N0, M0), IIB (T2, N1, M0 or T3, N0, M0) or IIIA (T1–2, N2, M0 or T3, N1–2, M0) were treated with NAC between 2007 and 2013. Tumour stage and T and N factors were stratified based on the TNM Classification of Malignant Tumours, UICC Seventh Edition (Ainbinder *et al*, 2009). Breast cancer was confirmed histologically using core needle biopsies and staged using systemic imaging studies employing computed tomography (CT), ultrasonography (US) and bone scintigraphy. Breast cancer was classified into subtypes according to the immunohistochemical expression of ER, PgR, HER2 and Ki67. Based on their immunohistochemical expression, the tumours are categorised into the immunophenotypes luminal A (ER+ and/or PgR+, HER2−, Ki67-low), luminal B (ER+ and/or PgR+, HER2+) (ER+ and/or PgR+, HER2−, Ki67-high), HER2-enriched (HER2BC) (ER−, PgR−, and HER2+) and TNBC (negative for ER, PgR and HER2). In this study, luminal A and luminal B were considered as hormone receptor-positive breast cancer (HRBC).

All patients received a standardised protocol of NAC consisting of four courses of FEC100 (500 mg m^−2^ fluorouracil, 100 mg m^−2^ epirubicin and 500 mg m^−2^ cyclophosphamide) every 3 weeks, followed by 12 courses of paclitaxel (80 mg m^−2^), administered weekly (Mauri *et al*, 2005; Mieog *et al*, 2007; Kawajiri *et al*, 2012). Forty-five patients were diagnosed with HER2-positive breast cancer and trastuzumab was administered on a weekly (2 mg kg^−1^) or tri-weekly (6 mg kg^−1^) basis, during paclitaxel treatment (Buzdar *et al*, 2007). All patients underwent chemotherapy as outpatients. Therapeutic antitumour effects were assessed according to the Response Evaluation Criteria in Solid Tumours (RECIST) criteria (Perez *et al*, 2013). Pathological complete response (pCR) was defined as the complete disappearance of the invasive compartment of the lesion with or without intraductal components, including the lymph nodes. Patients underwent mastectomy or breast-conserving surgery after NAC. All patients who underwent breast-conserving surgery were administered postoperative radiotherapy to the remnant breast. We operated on seven cases with progressive disease occurring during NAC treatment. Overall survival (OS) was defined as the period from the initiation of NAC to the time of death from any cause. Disease-free survival (DFS) was defined as the period in years, from the date of the primary surgery to the first local recurrence, distant recurrence or death from any cause. All patients were followed up with physical examinations every 3 months, US every 6 months and CT and bone scintigraphy annually. The median follow-up period for the assessment of OS was 3.4 years (range, 0.6–6.0 years) and 3.1 years for DFS (range, 0.1–6.0 years). One aspect of the study involved retrospective chart review. Written informed consent was obtained from all subjects. This research conformed to the provisions of the Declaration of Helsinki adopted in 1995. All patients were informed of the investigational nature of this study and provided their written, informed consent. The Ethics Committee of Osaka City University approved the study protocol (#926).

### Immunohistochemistry

All patients had a core needle biopsy prior to receiving NAC, and following treatment underwent either a mastectomy or breast-conserving surgery with axillary lymph node dissection at Osaka City University. Immunohistochemical studies on core needle biopsy specimens were performed as previously described (Kashiwagi *et al*, 2013). Tumour specimens were fixed in 10% formaldehyde solution and embedded in paraffin, and 4-*μ*m-thick sections were mounted onto glass slides. Slides were deparaffinised in xylene and heated for 20 min in Target Retrieval Solution (Dako, Carpinteria, CA, USA) in an autoclave (105 °C, 0.4 kg m^−2^). Specimens were then incubated with 3% hydrogen peroxide in methanol for 15 min to block the endogenous peroxidase activity, and then incubated in 10% normal goat or rabbit serum to block non-specific reactions.

Primary monoclonal antibodies directed against ER (clone 1D5, dilution 1 : 80; Dako), PgR (clone PgR636, dilution 1 : 100; Dako), HER2 (HercepTest; Dako), Ki67 (clone MIB-1, dilution 1 : 00; Dako) and AR (clone AR441, dilution 1 : 100; Dako) were used. Tissue sections were incubated with each antibody for 70 min at room temperature or overnight at 4 °C, and then incubated with horseradish peroxidase-conjugated anti-rabbit or anti-mouse IgG secondary antibodies (HISTOFINE (PO) kit; Nichirei, Tokyo, Japan). Slides were subsequently treated with a streptavidin-peroxidase reagent and incubated in phosphate-buffered saline-diaminobenzidine and 1% hydrogen peroxide (v/v), followed by counterstaining with Mayer's haematoxylin. Positive and negative controls for each marker were used according to the supplier's data sheet.

### Immunohistochemical scoring

**Figure 1:**
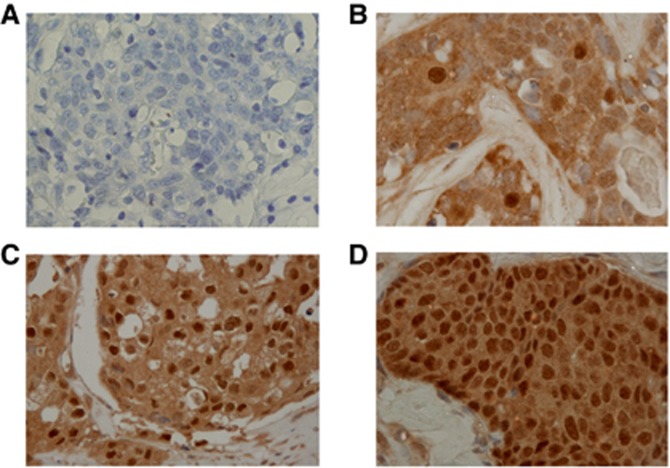
**Immunohistochemical analysis of androgen receptor (AR) expression.** AR expression was semiquantitatively analysed according to the percentage of cells showing positive expression in the nucleus: 0, 0% (**A**); 1+, 1–29% (**B**); 2+, 30–69% (**C**); 3+, ⩾70% (**D**). Androgen receptor expression was considered positive when scores were ⩾1, and negative when scores were 0.

Immunohistochemical scoring was performed by two pathologists specialised in mammary gland pathology, using the blind method to confirm the objectivity and reproducibility of each diagnosis. The cut-off value for ER and PgR positivity was set at ⩾1% in accordance with previous studies (Umemura *et al*, 2006), and the same cut-off was also adopted for AR positivity (Castellano *et al*, 2010; Luo *et al*, 2010). HER2 expression was scored according to the accepted grading system (0, no reactivity or membranous reactivity in less than 10% of cells; 1+, faint/barely perceptible membranous reactivity in ⩾10% of cells or reactivity in only part of the cell membrane; 2+, weak to moderate complete or basolateral membranous reactivity in ⩾10% of tumour cells; or 3+, strong complete or basolateral membranous reactivity in ⩾10% of tumour cells). HER2 expression was considered positive if the immunostaining score was 3+, or in cases where the score was 2+, gene amplification was determined via fluorescent *in situ* hybridisation (FISH). For FISH analysis, each copy of the *HER2* gene and its centromere 17 (CEP17) reference were counted. Interpretation of the results followed the criteria of the ASCO/CAP guidelines for HER2 IHC classification for breast cancer: positive if the HER2/CEP17 ratio was >2.0 (Wolff *et al*, 2013, 2014). A Ki67-labelling index of ⩾14% was classified as positive (Goldhirsch *et al*, 2011). Androgen receptor expression was semiquantitatively analysed according to the percentage of cells showing positive staining in the nucleus: 0, 0% 1+, 1–29% 2+, 30–69% 3+, ⩾70%. Androgen receptor expression was considered positive when scores were ⩾1, and negative when scores were 0 (Figure 1).

### Statistical analysis

Statistical analysis was performed using the SPSS version 19.0 statistical software package (IBM, Armonk, NY, USA). Categorical data are reported with numbers and percentages, and continuous data as a median and range. The association between AR and other clinicopathologic variables, and the significance of different prognostic markers were analysed using the chi-squared test (or Fisher's exact test when necessary). The association with survival was analysed using the Kaplan–Meier plot and log-rank test. The Cox proportional hazards model was used to compute univariable and multivariable hazard ratios (HR) for the study parameters with 95% confidence intervals, and used in a backward stepwise method for variable selection in multivariate analysis. In all of the tests, a *P*-value of less than 0.05 was considered statistically significant. Cut-off values for different biomarkers included in this study were chosen before statistical analysis.

## Results

The breast cancer subtypes of the 177 patients who received NAC were as follows: 61 (34.5%) were TNBC, 36 (20.3%) were HER2BC and 80 (45.2%) were HRBC (non-TNBC; HER2BC and HRBC). Androgen receptor expression was observed in 126 cases (71.2%). Among the 61 cases of TNBC, AR expression was positive in 23 cases (37.7%) and negative in 38 cases (62.3%). Among the 116 cases of non-TNBC, AR expression was positive in 103 cases (88.8%) and negative in 13 cases (11.2%) (HER2BC, positive in 30 cases and negative in 6 cases; HRBC, positive in 73 cases and negative in 7 cases). Androgen receptor expression was more commonly observed in patients with non-TNBC (*P*<0.001) and HRBC (*P*<0.001), but was not more commonly observed in patients with HER2BC (*P*=0.071). There was no significant difference in relation to other factors including pCR (*P*=0.108) for all breast cancer subtypes (Table 1). In contrast, analysis of the 61 TNBC cases revealed that the rate of pCR was significantly lower for patients in the AR-positive group (*P*=0.001; Table 2). However, there was no significant difference between AR and pCR in HER2BC (*P*=0.052) and HRBC (*P*=0.056) patients.

**Figure 2:**
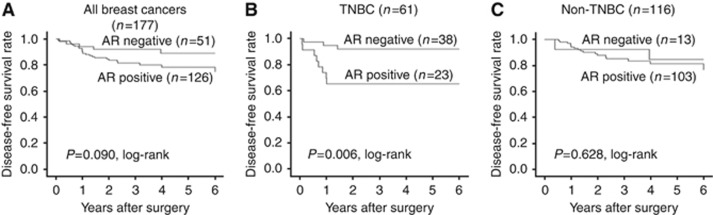
**Analysis of all 177 patients treated with neoadjuvant chemotherapy (NAC) revealed no significant difference in disease-free survival (DFS) associated with AR expression (*P*=0.090, log-rank) (**A**).** No significant difference in DFS according to AR expression was observed in patients with non-TNBC (*P*=0.628, log-rank) (**B**). A significantly shortened non-recurrence period was observed in patients with AR-expressing tumours, when the analysis was limited to patients with TNBC (*P*=0.006, log-rank) (**C**).

**Figure 3:**
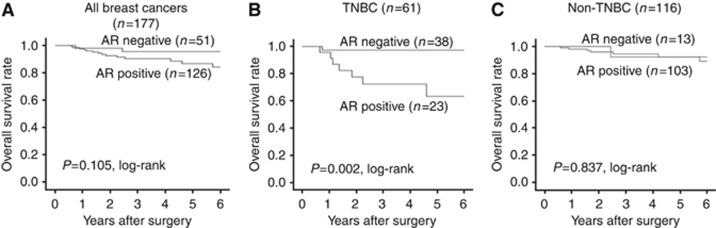
**Analysis of overall survival (OS) revealed similar observations according to the subtypes and AR expression status of the breast cancer (**A**–**C**).**

Analysis of all 177 patients receiving NAC revealed that no significant difference in DFS was associated with AR expression (*P*=0.090, log-rank; Figure 2A). Similarly, no significant difference in DFS was associated with AR expression in patients with non-TNBC (*P*=0.628, log-rank; Figure 2B). However, a significantly shortened non-recurrence period was observed in patients with AR-expressing tumours, when the analysis was limited to patients with TNBC (*P*=0.006, log-rank; Figure 2C). Analysis of OS demonstrated similar observations according to the subtypes and AR expression status of the breast cancer (Figure 3A–C). In univariate analysis, negative AR expression made a significant contribution to extending DFS in patients with TNBC (*P*=0.014, HR=5.26). However, multivariate analysis revealed that negative AR expression was not an independent factor (*P*=0.080, HR=3.78; Table 3).

Overall, 11 patients experienced recurrence events among the 23 patients with AR-positive TNBC (Table 4). The disease-free intervals were 0.10–1.42, with a median of 0.76 years. In three patients, recurrence occurred in the lung and liver (two cases). The remaining eight cases experienced disease recurrence either in the axillary lymph nodes or at the chest wall. Tissue samples of recurrent tumours were obtained from eight patients, and AR expression was confirmed in only two cases. Androgen receptor expression was not detected in the three recurrent foci previously shown to be negative in their surgical specimens.

## Discussion

Triple-negative breast cancer is typically associated with poor prognosis, based on the limited treatment options and the biological attributes of TNBC itself. At present, the NCCN guidelines and St Gallen Consensus conference also consider chemotherapy to be the only alternative for treatment. Despite this, a significantly higher rate of pCR is observed in TNBC patients following treatment with NAC compared with HRBC patients, with reports also suggesting that patients achieving pCR also demonstrated an extended period of non-recurrence (Masuda *et al*, 2013). Thus, in patients with TNBC, pCR is considered a useful surrogate marker to indicate the outcome of NAC. In this study, we observed that patients with AR-expressing TNBC had significantly lower rates of pCR, more common recurrence events and poorer prognosis than those with AR-negative TNBC. Our findings were in line with previous observations, suggesting that AR-positive TNBC is chemoresistant (Gucalp and Traina, 2010; Gucalp *et al*, 2013).

We observed frequent disease recurrence in patients with AR-positive TNBC. The disease-free intervals in these cases were very short, showing the typical pattern of recurrence in TNBC. We also observed frequent loss of AR expression in postoperative specimens after NAC, or in the recurrent foci of AR-positive TNBC in these patients, which might be due to the genetically unstable nature of TNBC during disease progression (Wang *et al*, 2014) or to accelerated genetic instability following NAC.

In our previous study, we demonstrated favourable prognosis in patients with AR-positive TNBC compared with those with AR-negative TNBC, following standard therapeutic strategies. Similar observations have been demonstrated in other studies (Moinfar *et al*, 2003; Gucalp *et al*, 2013). The present study demonstrates a different outcome in patients with AR-positive TNBC following NAC. In preclinical studies, it was reported that E-cadherin expression was inversely related to AR expression in TNBC (Graham *et al*, 2010). E-cadherin expression was reduced by AR expression in TNBC, and epithelial–mesenchymal transition (EMT) was induced and may show drug resistance.

Recent research indicates that TNBC can be categorised into seven specific subcategories based on genetic expression profiles (Lehmann *et al*, 2011; Metzger-Filho *et al*, 2012; Masuda *et al*, 2013). These include basal-like 1, basal-like 2, immunomodulatory, mesenchymal, mesenchymal-stem-like, luminal androgen receptor (LAR) and unstable cluster. Luminal androgen receptor is a subtype in which androgen signalling plays an important role in the cancer cell growth and progression (Gucalp and Traina, 2010). Furthermore, AR-positive TNBC has been reported to have similar genetic expression profiles compared with ER-positive breast cancer, and new, individually tailored treatments such as anti-androgen preparations that are currently in phase II testing are expected to show clinical benefit (Gucalp *et al*, 2013).

Given the results of this study, which indicate that AR-positive TNBC has low sensitivity to chemotherapy, AR-targeted-endocrine therapy may be one such promising therapeutic strategy. It is also known that AR and ER interact. Panet-Raymond *et al* (2000) demonstrated that the transcriptional activity of AR was inhibited by ER *in vitro*. In hormone-dependent breast cancer cells, induction of *7βHSD2* mRNA by active androgen dihydrotestosterone was inhibited by oestradiol (Takagi *et al*, 2010). These reports indicate insufficient AR activation by androgen in the presence of oestrogen, even in AR-positive cases, suggesting that AR-positive TNBC is an optimal candidate for antiandrogen therapy. In this study, we investigated changes in AR expression among AR-positive TNBC recurrences, and found that in many cases, AR expression in the tissue was lost at recurrence, indicating that AR expression was an inhibitory factor in TNBC.

The aim of this study was to clarify, based on AR expression in patients with breast cancer, the differences in chemosensitivity, clinically and pathologically. The pCR rate was significantly higher in AR-negative than in AR-positive patients with TNBC. Our study demonstrates that AR-negative TNBC patients receiving NAC under standardised protocols consisting of FEC, followed by weekly paclitaxel, do not show any survival benefit compared with patients with AR-positive TNBC. Moreover, induction of a change in subtype to the AR-negative phenotype was observed after NAC.

## Figures and Tables

**Table 1:** Correlation between clinicopathological features and androgen receptor expression in 177 breast cancers

	**AR expression in all breast cancers (*****n*****=177)**		
**Parameters**	**Positive (*****n*****=126)**	**Negative (*****n*****=51)**	***P*****-value**
**Age at operation**			
⩽56	64 (50.8%)	23 (45.1%)	
>56	62 (49.2%)	28 (54.9%)	0.492
**Menopause**			
Negative	52 (41.3%)	20 (39.2%)	
Positive	74 (58.7%)	31 (60.8%)	0.801
**Tumour size**			
⩽2 cm	18 (14.3%)	6 (11.8%)	
>2 cm	108 (85.7%)	45 (88.2%)	0.657
**Lymph node status**			
Negative	33 (26.2%)	8 (15.7%)	
Positive	93 (73.8%)	43 (84.3%)	0.134
**Nuclear grade**			
1, 2	98 (77.8%)	39 (76.5%)	
3	28 (22.2%)	12 (23.5%)	0.851
**Ki67**			
⩽14%	60 (47.6%)	14 (27.5%)	
>14%	66 (52.4%)	37 (72.5%)	0.014
**Intrinsic subtype**			
TNBC	23 (18.3%)	38 (74.5%)	
Non-TNBC	103 (81.7%)	13 (25.5%)	<0.001
**Intrinsic subtype**			
HER2BC	30 (23.8%)	6 (11.8%)	
Non-HER2BC	96 (76.2%)	45 (88.2%)	0.071
**Intrinsic subtype**			
HRBC	73 (57.9%)	7 (13.7%)	
Non-HRBC	53 (42.1%)	44 (86.3%)	<0.001
**Pathological response**			
pCR	43 (34.1%)	24 (47.1%)	
Non-pCR	83 (65.9%)	27 (52.9%)	0.108

Abbreviations: AR=androgen receptor; TNBC=triple-negative breast cancer; HER2BC=human epidermal growth factor receptor 2-enriched breast cancer; HRBC=hormone receptor-positive breast cancer; pCR=pathological complete response.

**Table 2:** Correlations between androgen receptor expression and clinicopathological parameters in 61 triple-negative, 36 HER2 enriched and 80 luminal type breast cancers

	**TNBC (*****n*****=61)**			**HER2BC (*****n*****=36)**			**HRBC (*****n*****=80)**		
**Parameters**	**Positive (*****n*****=23)**	**Negative (*****n*****=38)**	***P*****-value**	**Positive (*****n*****=30)**	**Negative (*****n*****=6)**	***P*****-value**	**Positive (*****n*****=73)**	**Negative (*****n*****=7)**	***P*****-value**
**Age at operation**									
⩽56	10 (43.5%)	15 (39.5%)		14 (46.7%)	4 (66.7%)		40 (54.8%)	4 (57.1%)	
>56	13 (56.5%)	23 (60.5%)	0.195	16 (53.3%)	2 (33.3%)	0.371	33 (45.2%)	3 (42.9%)	0.905
**Menopause**									
Negative	10 (43.5%)	12 (31.6%)		12 (40.0%)	5 (83.3%)		30 (41.1%)	3 (42.9%)	
Positive	13 (56.5%)	26 (68.4%)	0.348	18 (60.0%)	1 (16.7%)	0.133	43 (58.9%)	4 (57.1%)	0.928
**Tumour size**									
⩽2 cm	1 (4.3%)	6 (15.8%)		10 (33.3%)	0 (0.0%)		7 (9.6%)	0 (0.0%)	
>2 cm	22 (95.7%)	32 (84.2%)	0.174	20 (66.7%)	6 (100.0%)	0.096	66 (90.4)	7 (100.0%)	0.391
**Lymph node status**									
Negative	6 (26.1%)	5 (13.2%)		14 (46.7%)	2 (33.3%)		13 (17.8%)	1 (14.3%)	
Positive	17 (73.9%)	33 (86.8%)	0.203	16 (53.3%)	4 (66.7%)	0.549	60 (82.2%)	6 (85.7%)	0.815
**Nuclear grade**									
1, 2	16 (69.6%)	28 (73.7%)		21 (70.0%)	5 (83.3%)		61 (83.6%)	6 (85.7%)	
3	7 (30.4%)	10 (26.3%)	0.728	9 (30.0%)	1 (16.7%)	0.506	12 (16.4%)	1 (14.3%)	0.883
**Ki67**									
⩽14%	9 (39.1%)	9 (23.7%)		20 (66.7%)	3 (50.0%)		31 (42.5%)	2 (28.6%)	
>14%	14 (60.9%)	29 (76.3%)	0.200	10 (33.3%)	3 (50.0%)	0.438	42 (57.5%)	5 (71.4%)	0.476
**Pathological response**									
pCR	4 (17.4%)	24 (63.2%)		13 (43.3%)	0 (0.0%)		26 (35.6%)	0 (0.0%)	
Non-pCR	19 (82.6%)	14 (36.8%)	0.001	17 (56.7%)	6 (100.0%)	0.052	47 (64.4%)	7 (100.0%)	0.056

Abbreviations: AR=androgen receptor; TNBC=triple-negative breast cancer; HER2BC=human epidermal growth factor receptor 2-enriched breast cancer; HRBC=hormone receptor-positive breast cancer; pCR=pathological complete response.

**Table 3:** Univariable- and multivariable analysis with respect to disease-free survival in 61 triple-negative breast cancers

	**Univariable analysis**			**Multivariable analysis**		
**Parameter**	**Hazard ratio**	**95% CI**	***P*****-value**	**Hazard ratio**	**95% CI**	***P*****-value**
Lymph node status Negative *vs* Positive	0.94	0.20–0.94	0.939	1.15	0.25–5.44	0.857
Pathological response pCR *vs* non-pCR	0.23	0.05–1.08	0.063	0.45	0.08–2.46	0.356
Androgen receptor Positive *vs* Negative	5.26	1.39–19.86	0.014	3.78	0.85–16.68	0.080

Abbreviations: pCR=pathological complete response; CI.=confidence interval.

**Table 4:** The clinical background of 11 patients with AR-positive TNBC recurred

**No**	**Age**	**Menopause**	**Tumour size (cm)**	**Lymph node status**	**Nuclear grade**	**Ki67 (%)**	**Pathological response**	**Disease-free interval (years)**	**AR expression of CNB specimens before NAC**	**AR expression of surgical specimens after NAC**	**AR expression of CNB specimens after recurrence**
1	53	Positive	2.3	Positive	1	10	Non-pCR	0.85	Positive	Positive	Positive
2	40	Negative	3.2	Negative	3	75	pCR	0.76	Positive	NA	Positive
3	39	Negative	3.1	Positive	3	67	Non-pCR	0.99	Positive	Positive	Negative
4	30	Negative	3.3	Negative	2	50	Non-pCR	0.70	Positive	Positive	Negative
5	62	Positive	2.9	Positive	1	7	Non-pCR	0.10	Positive	Positive	Negative
6	65	Positive	2.5	Positive	3	71	Non-pCR	1.01	Positive	Positive	NA
7	58	Positive	2.2	Positive	2	57	Non-pCR	0.63	Positive	Positive	NA
8	44	Negative	1.8	Positive	2	82	Non-pCR	0.54	Positive	Negative	Negative
9	68	Positive	1.6	Positive	1	12	Non-pCR	0.88	Positive	Negative	Negative
10	63	Positive	3.8	Positive	3	62	Non-pCR	0.13	Positive	Negative	Negative
11	46	Negative	2.8	Positive	1	12	pCR	1.42	Positive	NA	NA

Abbreviations: AR=androgen receptor; CNB=core-needle biopsy; NAC=neoadjuvant chemotherapy ; pCR=pathological complete response; NA=not available.
